# Benefits and limitations of a new genome‐based PCR‐RFLP genotyping assay (GB‐RFLP): A SNP‐based detection method for identification of species in extremely young adaptive radiations

**DOI:** 10.1002/ece3.8751

**Published:** 2022-03-23

**Authors:** Claudius F. Kratochwil, Andreas F. Kautt, Sina J. Rometsch, Axel Meyer

**Affiliations:** ^1^ 26567 Zoology and Evolutionary Biology Department of Biology University of Konstanz Konstanz Germany; ^2^ Present address: 3835 Institute of Biotechnology HiLIFE University of Helsinki Helsinki Finland; ^3^ Present address: Department of Organismic and Evolutionary Biology Harvard University Cambridge Massachusetts USA

**Keywords:** Cichlidae, GB‐RFLP, genetic markers, Midas cichlids, species identification, targeted genome sequencing

## Abstract

High‐throughput DNA sequencing technologies make it possible now to sequence entire genomes relatively easily. Complete genomic information obtained by whole‐genome resequencing (WGS) can aid in identifying and delineating species even if they are extremely young, cryptic, or morphologically difficult to discern and closely related. Yet, for taxonomic or conservation biology purposes, WGS can remain cost‐prohibitive, too time‐consuming, and often constitute a “data overkill.” Rapid and reliable identification of species (and populations) that is also cost‐effective is made possible by species‐specific markers that can be discovered by WGS. Based on WGS data, we designed a PCR restriction fragment length polymorphism (PCR‐RFLP) assay for 19 Neotropical Midas cichlid populations (*Amphilophus* cf. *citrinellus*), that includes all 13 described species of this species complex. Our work illustrates that identification of species and populations (i.e., fish from different lakes) can be greatly improved by designing genetic markers using available “high resolution” genomic information. Yet, our work also shows that even in the best‐case scenario, when whole‐genome resequencing information is available, unequivocal assignments remain challenging when species or populations diverged very recently, or gene flow persists. In summary, we provide a comprehensive workflow on how to design RFPL markers based on genome resequencing data, how to test and evaluate their reliability, and discuss the benefits and pitfalls of our approach.

## Graphical abstract

Whole‐genome resequencing or even reduced representation sequencing remains costly and time‐consuming, if the only goal is species assignment. Here, we tested, if we can design a “traditional” PCR restriction fragment length polymorphism assay based on the genomic information at hand. We show that using this novel approach, that we coin “genome‐based PCR‐RFLP genotyping assay” (GB‐RFLP), the identification of species and populations can be greatly improved.

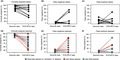

## 1 INTRODUCTION

Historically, morphological differences remain the basis for species identification, taxonomic keys, and effort in species delimitation. Yet, reliable classification of specimens can be complex due to many factors. For example, when species are morphologically extremely similar or when morphological characters are not expressed at a given life‐history stage (e.g., juveniles). In the last decade, the increasing affordability of reduced‐representation data (e.g., restriction site‐associated DNA sequencing or target enrichment) or whole‐genome resequencing has provided new possibilities to assign species not only based on morphological or meristic characters but also based on genomic information. In some instances, this has even greatly contributed to the discovery and description of new (i.e., previously cryptic) species (Fennessy et al., 2016; Nater et al., 2017). Genetic species assignment approaches are also promising to add novel tools to aid in conservation efforts of endangered species, but practical implementations often fail (Campbell et al., 2019; Piertney, 2016; Shafer et al., 2015). A major disadvantage of high‐throughput sequencing techniques are the cost and time that are needed to generate libraries, sequence them, and analyze the data. But, importantly, genomic data also allow for the identification of a suite of informative, diagnostic genetic markers for species or population assignment that can be genotyped using cheaper and faster methods (Shafer et al., 2015).

Among all genetic variants, single‐nucleotide polymorphisms (SNPs) are clearly the most abundant (in the human population, e.g., more than 95% of all genetic variants are SNPs (Auton et al., 2015)) and, therefore, powerful genetic markers for assigning populations or species. Over the past 30 years, many methods have been developed to cost‐effectively genotype SNPs. One widely used, fast method is PCR restriction fragment length polymorphism (PCR‐RFLP) (McKeown et al., 2015; Ota et al., 2007). Hereby, a particular DNA fragment is first amplified by PCR. The resulting amplicon is then digested using a restriction enzyme that cuts only one allele at a diagnostic SNP (resulting in two fragments) but not the other one (one fragment), due to an ideally species‐specific polymorphism in the enzyme's recognition site. Homozygous individuals for either allele, as well as heterozygous individuals (three fragments), can be easily distinguished from each other by gel electrophoresis (see detailed description of the method in Ota et al., 2007). Therefore, PCR‐RFLP is an excellent method that can be used for fast, cheap, and reliable genotyping of diagnostic markers.

Recently, we have sequenced 453 genomes of a very young species flock of Nicaraguan Midas cichlid fishes (*Amphilophus cf*. *citrinellus*) (Kautt et al., 2020). This species complex includes, so far, 13 described species (Torres‐Dowdall & Meyer, 2021). Two species (*A*.*s citrinellus* and *A*. *labiatus*) can be found in both Great Lakes Managua and Nicaragua (Barluenga et al., 2006). From there, seven crater lakes (Apoyeque, Apoyo, As. León, As. Managua, Masaya, Tiscapa, and Xiloá) have been colonized (Elmer et al., 2009, 2013, 2014). In two of the crater lakes, Apoyo and Xiloá, six and four endemic species have been described, respectively (Barlow & Munsey, 1976; Geiger et al., 2010; Recknagel et al., 2013; Stauffer Jr et al., 2008; Stauffer Jr & McKaye, 2002). In crater lake As. Managua, another endemic species, *A*. *tolteca*, has been formally described (Recknagel et al., 2013), while species of the other crater lakes await formal description (why we included them here as “populations”).

Crater lake populations and sympatric species therein clearly form separate clusters using both RAD‐sequencing data (Kautt et al., 2018) and whole‐genome data (Kautt et al., 2020). While all crater lake populations and species differ morphologically (Elmer et al., 2010; Kautt et al., 2018), species assignment can be difficult, especially when specimens are young, and particularly for the sympatric species from crater lakes Apoyo and Xiloá. Therefore, methods to quickly genotype fish using genetic markers would give additional confidence for species assignments and allow identification of species also for juvenile fish. This is important for certain research questions including, for example, cohort analyses and unbiased frequency estimations. Moreover, several of these species are protected or live in protected environments where illegal fishing occurs. Cheap genotyping assays with a fast turnaround time might contribute to conservation monitoring.

The objectives of this study were, therefore, to (1) design a workflow to screen for suitable GB‐RFLP markers for species and population assignment, (2) test in silico if those markers would allow unambiguous assignment, and (3) to perform GB‐RFLP assays on independent samples (i.e., samples that have been not used for the design of the markers in (1)) to test if the markers are suitable to assign species and populations (i.e., lakes of origin).

## 2 MATERIALS AND METHODS

### 2.1 Study system, samples, and genomic data

**FIGURE 1:**
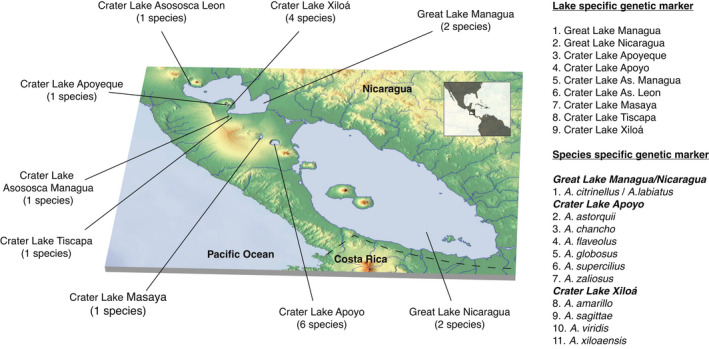
The Midas cichlid species complex includes fish inhabiting nine lakes (the two great lakes and seven crater lakes) and comprises 13 described species. In this study, we designed genome‐based PCR‐RFLP (GB‐RFLP) markers for population (lake‐specific genetic markers) and species assignment (species‐specific genetic markers)

The Midas cichlid species complex encompasses a total of—based on genetic clustering (Kautt et al., 2020)—at least 19 genetically distinguishable populations that include the 13 described species (Figure 1). These include five crater lakes with only one species (Lakes Apoyeque, As. León, As, Managua, Masaya, and Tiscapa), one crater lake with six species (Crater Lake Apoyo), one crater lake with four species (Crater Lake Xiloá), and the Great Lakes Managua and Nicaragua that both harbor the same set of two species (*A*. *citrinellus* and *A*. *labiatus*; Figure 1). To find species and population‐specific markers, we used genotype calls from a previous analysis of 453 resequenced Midas cichlid genomes, including all aforementioned species and lake populations (Kautt et al., 2020). Tissue samples were collected during field trips to Nicaragua between 2003 and 2018 (permit numbers DGRNB‐ACHL‐0078, DGRNB‐IC‐006‐2007, No. 026‐11007/DGAP y DGPN/DB‐27‐2010, DGPN/DB/DAP‐IC‐0003‐2012, DGPN/DB‐02‐2012, DGPN/DB‐IC‐004‐2013, DGPN/DB‐011‐2014, DGPN/DB‐IC‐015‐2015, DGPN/DB‐IC‐073‐2017, Ministerio del Ambiente y los Recursos Naturales (MARENA), Nicaragua).

### 2.2 Screen for species and population‐specific variants

**FIGURE 2:**
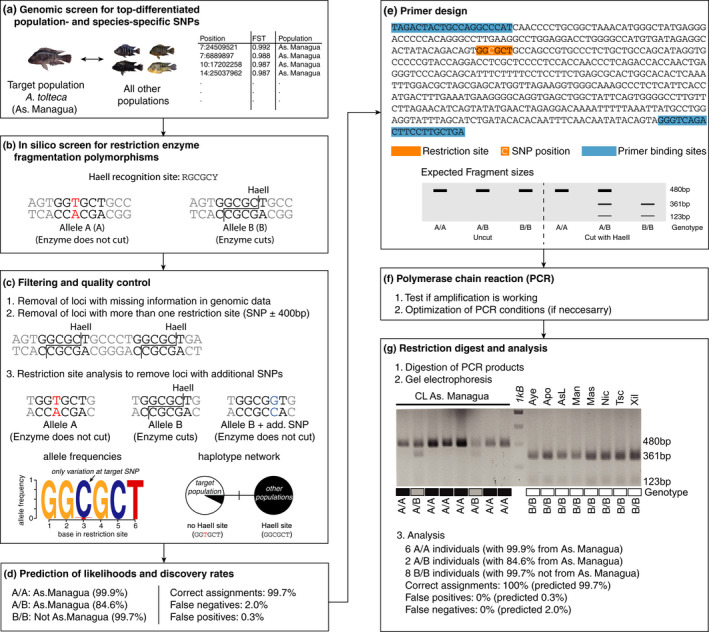
Workflow of the whole‐genome resequencing‐based design of PCR‐RFLP markers (GB‐RFLP). (a) Markers were designed based on genetic differentiation (*F*_ST_) or genome‐wide genotype‐phenotype association (GWA) data of a 453‐genome dataset using pairwise ingroup–outgroup comparisons. (b) Variants were screened for RFLPs with one allele (but not the other) being cut by a restriction enzyme. (c) Target variants were filtered based on the presence of additional restriction sites and additional SNPs within the restriction site. Quality control was performed by plotting allele frequencies and haplotype networks. (d) Likelihoods that a genotype corresponds to a population (or not) were calculated based on population‐specific allele frequencies. Percentage of correct assignments together with false negative and false positive rates were based on bootstrapping of genotypes (informed by empirical allele frequencies in the genomic dataset). (e) Primers were designed based on 801‐bp sequences (core SNP ± 400 bp) in a way that the restriction enzyme would generate two fragments with a ~1:2 length ratio. (f) PCR conditions were tested and optimized. (g) Restriction digest was performed on 8 ingroup samples and 5–8 outgroup samples. Genotypes were determined by visual inspection. These data were used to calculate the number of correct assignments as well as rates of false positives and negatives (as for the bootstrapping dataset in d)

To screen for species‐ and population‐specific markers, we estimated population differentiation (F_ST_) for individual SNP loci using *vcftools* (Danecek et al., 2011), always comparing the respective focal population (ingroup) to all other samples (outgroup) (the workflow is also described in Figure 2). For Crater Lakes Apoyo and Xiloá, we compared the focal species (ingroup) against all other sympatric species (outgroup). For the two great lake species, we used instead genome‐wide association (GWA) data for lip shape, which is a characteristic trait that differs between *A*. *citrinellus* and *A*. *labiatus* and correlates with F_ST_ in both lakes (Kautt et al., 2020). For each species and population, we extracted the 20 variants with the highest differentiation (*F*_ST_)/genome‐wide association (GWA).

### 2.3 Screen for RFLP alleles

To screen for RFLP alleles, we extracted 801 bp flanking the target variants (focal marker ± 400 bp) using samtools faidx from the *A*. *citrinellus* reference genome (Kautt et al., 2020). Genotypes for all loci were extracted from an in‐house filtered statistically phased variant call format (vcf) file (Kautt et al., 2020) using tabix. Nucleotide sequences and genotypes of variants (using *vcfR* (Knaus et al., 2020)) were loaded into R (R Development Core Team, 2019). For each locus, we generated a sequence with the alternative allele (alternative sequence)—for this initial analysis we did not use phased haplotypes. Next, we generated a list of the recognition sites of 237 commercially available restriction enzymes and performed in silico restriction digests of reference and alternative sequences for each locus. For each locus, we identified restriction enzymes cutting one of the alleles but not the other one and generated a list of all enzymes, including information on how many restriction sites could be found within the 801 bp reference sequence. For every locus, we then sorted the restriction sites by number of cutting sites within the 801 bp sequence.

### 2.4 Filtering and quality control

We filtered our genetic marker set in several ways. First, we removed variants that had missing data across the dataset and avoided markers that were on the same chromosome (to avoid that the markers are in linkage disequilibrium). Second, we selected 801‐bp loci with a single restriction sites (or a maximum of two for a few). Third, we avoided genetic markers that had other variants in the flanking region (±5 bp). Based on the position of the restriction enzyme recognition sequence, we extracted the exact haplotypes for all sequenced genomes, estimated allele frequencies across the dataset and constructed haplotype networks (Figures S1 and S2). Two RFLP loci had one additional SNP in the recognition sequence. However, these SNPs did not affect the RFLP. In one case, the second SNP was fully linked to the target SNP (Figure S2L). In the other case, there were two haplotypes that both were not cut by the enzyme and both were more common in the outgroup (Figure S1O).

### 2.5 Primer design

Primers were designed using Primer3 (Sequences are summarized in Table S1). Briefly, we used the 801‐bp sequence and designed the primers asymmetrically around the target SNP (i.e., that approximately one third (or two thirds) of the amplicon is 5’ of the target SNP) to avoid that the fragments have the same size after digestion. If the previous analysis indicated additional restriction sites in the 801 bp sequence, we confirmed that the restriction sites are either outside of the amplicon, or at least do not complicate the detection of the RFLP (e.g., for the Lake Managua marker Chr3:33,260,056 the alleles resulted in 402‐, 106‐, and 50‐bp or 448‐ and 106‐bp‐long fragments).

### 2.6 DNA extraction and PCR

DNA was extracted as described previously (Kratochwil & Rijli, 2014). Briefly, we incubated fin or muscle tissue for 2 h on a shaking incubator at 55°C using 200 µg/ml proteinase K (Sigma) in 500 µl extraction buffer (100 mM Tris–HCl pH 8.5, 200 mM NaCl, 5 mM EDTA, 0.2% SDS) in 1.5‐ml tubes. Tubes were centrifuged at 12,000–16,000 ×g and 500 µl isopropanol was added to the supernatant and centrifuged again. The supernatant was removed, and the pellet was washed with 500 µl of 70% ethanol. The pellet was air dried and diluted in 500 μl 10 mM Tris‐Cl, pH 8.5. We did not use EDTA as it might affect restriction enzyme activity. PCRs were performed for eight samples (with the exception of a few cases where we did not have enough individuals) of the target population (ingroup) and 5–8 samples that did not belong to the target population (outgroup). For the outgroups, we tried to use representatives of each lake (for the lake comparisons, i.e., population comparisons) or other species living in sympatry (for the species comparisons). We did not include samples that were part of the previously generated genome resequencing dataset. For PCRs, we used 2µl template and the standard protocol of DreamTaq DNA Polymerases (Thermo Fisher) with a total volume of 20µl, an annealing temperature of 58°C, 30 cycles, and 30‐s extension time.

### 2.7 PCR‐RFPL analysis

A volume of 17 µl of the PCR amplicons was digested using the respective restriction enzymes (AccI, AciI, AleI, AlwNI, ApoI, AvaI, BbvI, BccI, BceAI, BclI, BsaAI, BsaBI, BsaI, BsiEI, BsmI, BspMI, BsrI, BstAPI, HaeII, HaeIII, HgaI, HinfI, HpaII, HphI, NcoI, NspI, PleI, PstI, PvuII, RsaI, Tth111I, XcmI; all from New England Biolabs) and recommended concentrations. PCR products were digested for 4h or overnight to avoid partial digestion and loaded on a 1% agarose gel. Genotypes were determined by visual inspection of gels and gel photographs (Figures S3 and S4). Sometimes, despite long digestion time, we observed incomplete digestion. Genotypes were assigned as follows: (1) A lack of a smaller fragment was always interpreted as a homozygous genotype. (2) A band at the position of the uncut amplicon together with a second band was interpreted as a heterozygous genotype, but only if the uncut band was stronger in intensity (as the same number of longer sized DNA molecules results in a stronger signal). (3) Complete lack of a band at the size of the uncut fragment or a band that had less intensity than the digested fragments were interpreted as a homozygous genotype for the alternative allele.

### 2.8 Estimation of RFLP marker quality

To estimate the reliability of individual markers, we first calculated discovery and false discovery rates based on allele frequencies. To do so, we first calculated allele frequencies by lake (lake comparisons) or sympatric species (species comparisons). Based on the allele frequency, we calculated expected genotype frequencies for all populations individually (assuming Hardy–Weinberg equilibrium) (Table S2). To evaluate the quality of the markers, we used these frequencies to calculate the chance that an individual with a particular genotype is from a particular population or not (without specifying which and assuming a 50:50 chance that the individual is from the focal population or not) (Table S2).

**FIGURE 3:**
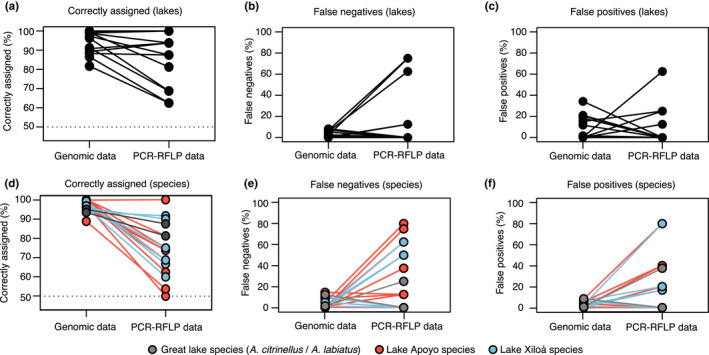
Comparison of the percentage of correctly assigned samples (a, d), false negatives (b, e), and false positives (c, f) using lake‐specific GB‐RFLPs (a–c) and species‐specific GB‐RFLPs (d–f). On the left side of the plots are always estimates based on bootstrapping data using allele frequencies from a previously generated genomic dataset, data from GB‐RFLP analyses are on the right. Color code in d–f indicates the populations of Crater Lake Apoyo (red), Xiloá (blue), and the great lake species *A*. *citrinellus* and *A*. *labiatus* (gray)

We used a bootstrap approach and randomly picked one million genotypes (i.e., individuals) from the ingroup (target population) and one million from the outgroups (again with an equal chance for each population/sympatric species to be picked) based on their relative frequencies and calculated how often a particular population/sympatric species would have been assigned correctly (correctly assigned), how often an ingroup individual would have been assigned to an outgroup (false negative) and how often an outgroup individual would have been assigned to the ingroup (false positive) (Figure 3, Table S3). The same approach was then used based on our PCR‐RFPL data (Table S3): False negatives were ingroup individuals that were incorrectly assigned as outgroup individuals, false positives were outgroup individuals that were incorrectly assigned as ingroup individuals. The proportion of correctly assigned individuals was calculated by taking the mean of the percentage of correctly assigned ingroup and the percentage of correctly assigned outgroup individuals (to make these estimates comparable to the estimates based on the bootstrapping dataset—some analyses were imbalanced with a different number of ingroup and outgroup individuals).

## 3 RESULTS

### 3.1 GB‐RFLP marker analysis for lake of origin assignment

As a first step, we chose RFLP markers that are specific for lakes (i.e., allowing to distinguish one population [ingroup] from all other populations [outgroup]) (Figures 1 and 2). Although not all of them are (yet) described as separate species, genetic differentiation is very high among most of the lake populations—mainly because the crater lakes are completely isolated (and therefore not permitting gene flow) and because their founder populations were small (30–850 individuals), resulting in a strong genetic drift (and thereby more alternatively fixed alleles) (Kautt et al., 2020). Most variants have likely been recruited from the standing genetic variation present in the source populations that was introduced into the crater lakes with the colonizers, as there was too little time for a substantial amount of *de novo* mutations to occur—all crater lakes have been colonized <5000 years ago (Kautt et al., 2016, 2018, 2020). The two Great Lakes Managua and Nicaragua are not isolated from each other, but intermittently connected by a river (Rio Tipitapa) resulting in limited ongoing gene flow (based on demographic models from (Kautt et al., 2020) for *A*. *citrinellus* approximately 1 in 12000 individuals per generation).

To assess the performance of markers, we calculated their predictive power (see detailed description in Materials and Methods section) based on bootstrapping using allele frequencies from a previous genomic study that was based on 453 resequenced genomes (Kautt et al., 2020), as well as GB‐RFLP assays of additional samples in this study (that were not part of the resequenced genomes from the (Kautt et al., 2020) study, but morphologically assigned to lakes and species). In line with the discussed potential effect of the described demographic parameters (Kautt et al., 2020), RFLP markers were most powerful for crater lakes. For ten out of the twelve RFLP markers for crater lakes Apoyeque (Figure S3E), Apoyo (Figure S3G, H), As. Managua (Figure S3I, J), As. León (Figure S3K, L), Masaya (Figure S3M, N), Tiscapa (Figure S3O, P), and Xiloá (Figure S3Q), more than 90% of the samples were correctly assigned to the lake (Table 1). The second Apoyeque marker (87%, Figure S3F) and the second Xiloá marker (69%, Figure S3R) performed less well. For the Great Lakes Managua (both 62%) and Nicaragua (87% and 69%), the percentage of correctly assigned samples was, as expected, lower (Figure S3A–D), as many variants are shared between the two great lakes or can be found at least in one of the crater lakes (as they have been colonized from the great lakes).

**TABLE 1:** Tested RFLP markers, their location in the reference genome, used restriction enzyme, quality of the marker, and correctly assigned individuals (in %)

Marker (Test population | coordinates)	Enzyme	Marker quality	Genotype (% correctly assigned)	
			Ingroup	Outgroup
Lake Managua | Chr3:33,260,056	HaeII		402/402 (99.8%) 550/402 (96.3%)	550/550 (73.8%)
Lake Managua | Chr21:22,155,441	BclI	−	400/400 (98.9%) 529/400 (88.2%)	529/529 (82.1%)
Lake Nicaragua | Chr11:21,205,346	BsrI	+	395/395 (98.9%) 544/395 (86.7%)	544/544 (85.5%)
Lake Nicaragua | Chr17:561,899	BsaAI	−	400/400 (99.2%) 551/400 (89.2%)	551/551 (88.9%)
Lake Apoyeque | Chr13:8,711,066	PleI	++	607/607 (100%) 607/367 (51.3%)	367/367 (100%)
Lake Apoyeque | Chr11:1,877,014	PvuII	+	585/585 (99.7%) 585/363 (59.6%)	363/363 (99.9%)
Lake Apoyo | Chr18:5,677,584	HpaII	++++	523/523 (100%) 523/362 (59.4%)	362/362 (100%)
Lake Apoyo | Chr11:1,173,519	ApoI	++++	402/402 (100%) 533/402 (86.5%)	533/533 (100%)
Lake As. Managua | Chr7:6,889,897	HaeII	+++	480/480 (99.9%) 480/361 (84.6%)	361/361 (99.7%)
Lake As. Managua | Chr23:32,083,942	NcoI	+	196/196 (100%) 557/196 (100%)	557/557 (98.5%)
Lake As. León | Chr18:14,951,950	BsaI	++++	530/530 (100%)	405/405 (100%)
Lake As. León | Chr8:21,669,333	BceAI	++++	500/500 (100%)	500/188 (100%) 188/188 (100%)
Lake Masaya | Chr20:9,539,893	BccI	++	400/400 (100%) 553/400 (99.5%)	553/553 (84.7%)
Lake Masaya | Chr11:20,264,687	HaeIII	+	492/492 (100%) 492/363 (100%)	363/363 (82.4%)
Lake Tiscapa | Chr18:17,567,949	HphI	++++	411/411 (100%)	528/528 (100%)
Lake Tiscapa | Chr14:11,451,235	AvaI	++++	524/524 (100%) 524/401 (100%)	401/401 (99.8%)
Lake Xiloá | Chr3:13,299,069	BsaI	++++	543/543 (100%) 543/408 (97.1%)	408/408 (99.2%)
Lake Xiloá | Chr14:29,887,480	HgaI	−	506/506 (99.6%) 506/377 (63.4%)	377/377 (99.6%)
*A*. *citrinellus* / *A*. *labiatus* | Chr8:11,495,129	AccI	+	602/602 (98.5%)	602/351 (71%) 351/351 (99.8%)
*A*. *citrinellus* / *A*. *labiatus* | Chr8:11,381,985	NspI	+	348/348 (99.5%) 480/348 (57.4%)	480/480 (99.1%)
*A*. *astorquii* | Chr24:27,792,838	BsmI	−	370/370 (100%) 515/370 (96.8%)	515/515 (94.5%)
*A*. *astorquii* | Chr11:4,091,881	AlwNI	−	536/536 (99.4%) 536/389 (67%)	389/389 (99.2%)
*A*. *chancho* | Chr17:14,664,535	PstI	−	397/397 (99.9%)	520/397 (100%) 520/520 (100%)
*A*. *chancho* | Chr3:25,801,703	AciI	++++	112/112 (99.5%)	488/112 (100%) 488/488 (100%)
*A*. *flaveolus* | Chr15:22,314,952	ApoI	−	538/538 (95.2%)	538/185 (100%) 185/185 (100%)
*A*. *flaveolus* | Chr20:3,776,504	HinfI	−	524/524 (98.3%) 524/397 (74.7%)	397/397 (91.5%)
*A*. *globosus* | Chr3:19,696,124	BsaBI	−	536/536 (100%) 536/197 (100%)	197/197 (100%)
*A*. *globosus* | Chr12:23,301,195	HgaI	−	382/382 (100%) 517/382 (100%)	517/517 (99.4%)
*A*. *supercilius* | Chr2:17,623,789	RsaI	−	583/583 (100%) 583/199 (96.4%)	199/199 (96.1%)
*A*. *zaliosus* | Chr24:14,867,912	BbvI	+	531/531 (99.9%)	531/413 (100%) 413/413 (100%)
*A*. *amarillo* | Chr6:811,192	BsiEI	−	514/514 (100%) 514/169 (86.8%)	169/169 (99.5%)
*A*. *sagittae* | Chr18:24,041,527	BstAPI	++	535/535 (99.3%) 535/171 (71.7%)	171/171 (97.6%)
*A*. *viridis* | Chr5:15,701,465	XcmI	+	175/175 (100%) 563/175 (96.2%)	563/563 (93.4%)
*A*. *viridis* | Chr16:24,619,817	BspMI	−	566/566 (99.8%) 566/185 (82.3%)	185/185 (97.8%)
*A*. *xiloaensis* | Chr4:5,998,299	Tth111I	−	512/512 (100%) 512/155 (100%)	155/155 (98.5%)
*A*. *xiloaensis* | Chr8:24,801,784	AleI	−	593/593 (99.8%)	593/391 (53.4%) 391/391 (99.9%)

Note

Quality was assessed based on a combination of the predicted and tested number of correctly assigned specimens (++++: >99%, +++: >95%, ++: >90%, +: >80%, –: <80%). Ingroup means “within test population” and outgroup means “not within test population.” For lake markers (above line), the outgroup contains all samples, for species markers (below line) only sympatric species within the same respective crater lake.

Most markers showed similar accuracy as in the bootstrapping dataset that was based on allele frequencies in the respective lakes with 1/18 markers performing much worse (>20% fewer correctly assigned samples; Figure S3R) in the RT‐RFLP assay than in the bootstrapping dataset (Figure 3a–c). We also tested whether a combination of two markers would improve accuracy. For two populations, Great Lake Nicaragua (69% instead of 62.5%) and Crater Lake Masaya (100% instead of 94%), we found a marginal improvement of correct assignments (Table S4).

### 3.2 GB‐RFLP marker analysis for the assignment of sympatric crater lake species

Next, we designed markers to distinguish the sympatric species of Crater Lakes Apoyo (*A*. *astorquii*, *A*. *chancho*, *A*. *flaveolus*, *A*. *globosus*, *A*. *supercilius*, and *A*. *zaliosus*) and Xiloá (*A*. *amarillo*, *A*. *sagittae*, *A*. *viridis* and *A*. *xiloaensis*). Although all sympatric species clearly form separate genetic clusters, the number of shared alleles is extensive due to ongoing gene flow and/or recent divergence (Kautt et al., 2016, 2018). Indeed, there are no alternatively fixed differences, but we show that there are some nearly fixed ones based on which we should reach 90% to 100% accuracy; however, it is possible that these predictions might be limited because our genomic samples do not accurately enough reflect the actual population frequencies.

Overall, the quality of the species‐specific RFLP markers (Figure S4) was much lower than the lake‐specific markers (Figure S3). The percentage of correctly assigned species ranged from 50% (equal to a random assignment) to 100%. Only two markers achieved values above 90% (*A*. *chancho*, Figure S4F and *A*. *viridis*, Figure S4O). In contrast to the lake‐specific markers, species‐specific markers showed a much lower accuracy in the GB‐RFLP assay compared to the bootstrapping dataset with 12/16 samples having >20% less correctly assigned samples than in the bootstrapping dataset (Figure 3d–f), which is substantially different from the lake‐specific markers (1/18). The combination of markers did not improve the accuracy.

### 3.3 GB‐RFLP marker analysis for the assignment of the great lake species *A. citrinellus* and *A. labiatus*

Lastly, we designed markers for the two great lake species. As genetic differentiation between these species is very low (0.015–0.02) compared to the crater lake or allopatric species pairs (0.07–0.5), we used SNPs that we have identified based on genome‐wide association mapping for the species‐defining trait: lip size (*A*. *labiatus* has thick, hypertrophic lips that *A*. *citrinellus* lacks) (Kautt et al., 2020; Machado‐Schiaffino et al., 2017). As the phenotype is largely driven by a single locus, the variants were on the same chromosome (distance: 113 kb). For the two species‐specific markers that we designed, we predicted an accuracy of 92% and 99%, respectively. When tested, the GB‐RFLP markers obtained similar accuracies of 87% and 81%, respectively (Figure S4a, b and Figure 3d–f). Interestingly, even though both loci are in proximity on the same chromosome, the combination of both markers lead to an accuracy of 100%.

## 4 DISCUSSION

Despite the increase in large‐scale genomic data, PCR‐RFLPs are still widely used as diagnostic markers for the detection and species assignment of parasites (Pegg et al., 2016), disease‐causing pathogens (Kato et al., 2019), microbiota (Baffoni et al., 2013), toxic dinoflagellates (Lozano‐Duque et al., 2018), as well as animals using different tissue samples (Larraín et al., 2019), scat samples (Mukherjee et al., 2010), or environmental DNA (eDNA) (Clusa et al., 2017). Once markers are identified, it is a fast, cheap, and reliable technique, but the design of PCR‐RFLP markers is usually time consuming, especially if many species and populations are being compared and/or highly differentiated markers are difficult to find. Here, we introduce a streamlined workflow to identify PCR‐RFLPs from whole‐genome resequencing data (GB‐RFLPs). We note that the same approach could be applied to RAD‐seq, exome sequencing, or other forms of targeted genomic data (Figure 2).

Our study yielded promising results for diverged populations from different lakes without ongoing gene flow, as represented by all seven Nicaraguan crater lakes. While populations could be assigned with more than 90% accuracy to two crater lakes (Apoyeque and As. Managua), our markers even yielded 100% assignment accuracy for populations of the remaining five crater lakes (Table S4). Results were less clear for populations with ongoing gene flow and/or large population sizes, in particular, the Great Lakes Nicaragua and Managua, for which population‐specific markers performed poorly (between 62 and 86% assignment accuracy, Table S4). This was not unexpected as we know that many alleles are shared between the great lakes and chances are high that alleles found in one of the great lakes can at least be found in one of the crater lakes that was colonized from this older source population. Therefore, although we could assign individual samples using whole‐genome (Kautt et al., 2020) or RAD‐seq data (Kautt et al., 2018), single or double marker approaches are not sufficient to unambiguously differentiate between Lake Managua or Lake Nicaragua Midas cichlid populations. A similar problem can be observed for the species‐specific markers for the species of crater lakes Apoyo and Xiloá. Also, here, species clearly form pronounced clusters using whole‐genome (Kautt et al., 2020) or RAD‐seq marker sets (Kautt et al., 2016). Yet, particularly in the sympatric scenario, where speciation occurred within the last 5,000 years (Kautt et al., 2020) and, in at least one case, gene flow persists (Kautt et al., 2018, 2020), there might be a strong ascertainment bias when focusing on single SNPs—as it has been intensively discussed for SNP datasets from humans (Clark et al., 2005). In line with this caveat, indeed species‐specific markers, with a few exceptions (*A*. *chancho* and *A*. *viridis*), performed less reliably (12/14 markers have <90% correct assignments; Table S4). Interestingly, the genetic markers for the great lake species that show extremely low genetic differentiation (FST~0.02) perform quite well (87% and 81% correctly assigned), particularly when combined (100% correctly assigned) (Figure S4). This can be explained by the different approach that was taken here. We designed markers based on the cognizant of our prior knowledge of the genomic basis of the species‐defining trait of *A*. *labiatus*: hypertrophied, thick lips. As the trait and the underlying associated SNPs (lip size variation links to only a single locus in most populations; (Kautt et al., 2020)) are almost alternatively fixed between these species, the markers seem to be most powerful to reliably assign species. While signals for gene flow between *A*. *labiatus* and *A*. *citrinellus* can be detected in most of the genome, this is not true for the lip locus on chromosome 8, where the genetic markers are also located.

Based on our results, we conclude that the design of markers based on whole‐genome data is a powerful approach in an effort to distinguish clearly differentiated species or populations or rare cases where we have loci with high local differentiation that can be used as markers. For populations with ongoing gene flow or instances where the population constitutes the source population (both applies for Great Lakes Nicaragua and Managua), the single/double‐GB‐RFLP marker approach performs poorly—likely because our genomic samples that we used for the design of the markers give only an estimate of the “true” population allele frequencies (i.e., markers that seem perfect based on our limited genomic data are in reality not markers that can unambiguously differentiate populations). The same is true for sympatric species (Crater Lakes Apoyo and Xiloá) without localized differentiation (as opposed to differentiation found between *A*. *labiatus* and *A*. *citrinellus*). To make reliable species identification possible, multimarker assays might be necessary for some instances. These would likely not require the complete set of markers found via RAD‐seq or WGS analyses, but could be applied with a selected set of markers. Here, one approach would be to use those SNPs that load most heavily on the first principal components of Crater Lakes Apoyo and Xiloá (based on Kautt et al., 2016, 2018, 2020), thereby giving most power to distinguish the sympatric species. Such very cost‐effective targeted multi‐SNP genotyping panels have been used, for example, for 217 SNPs to assign salmons to particular populations (Aykanat et al., 2016) and might be an excellent approach, also for the Midas cichlid system. Lastly, this set of RFLPs is now available as a resource for conservation purposes to, for example, not only identify individual samples on fish markets but also for cohort and mark‐recapture studies. This study, therefore, also presents a workflow for how to use genomic resources for the generation of applicable low‐budget approaches for species assignment. Our study, therefore, introduces a new methodological approach for such an effort, as implementation of approaches that can help “real‐world conservation issues” often fail as previously discussed (Shafer et al., 2015).

In summary, we tested a set of 36 PCR‐RFLP loci that we designed based on whole‐genome resequencing data to genetically assign Midas cichlid species and populations. While our analyses reveal limitations for the assignment of species and populations with ongoing gene flow and/or extreme recent divergence, genome‐based designed PCR‐RFLPs (GB‐RFLP) have great benefits when populations with robust genome‐wide (allopatric populations) or local differentiation (*A*. *citrinellus* and *A*. *labiatus*) have to be identified.

## CONFLICT OF INTEREST

The authors declare that there is no conflict of interests.

## AUTHOR CONTRIBUTIONS

**Claudius F. Kratochwil:** Conceptualization (equal); Formal analysis (equal); Methodology (equal); Writing – original draft (lead). **Andreas F. Kautt:** Formal analysis (equal). **Sina J. Rometsch:** Methodology (equal); Writing – review & editing (equal). **Axel Meyer:** Conceptualization (equal); Writing – review & editing (equal).

## Supporting information

Supplementary Material

## Data Availability

All data to reproduce these results are part of the submitted manuscript. The genomic data this study is based on have been previously described in Kautt et al. (2020) and have been deposited at DDBJ/ENA/GenBank under accession JACBYM000000000 and PRJEB38173.
