# Supplementary material for: Benefits and limitations of a new genome‐based PCR‐RFLP genotyping assay (GB‐RFLP): A SNP‐based detection method for identification of species in extremely young adaptive radiations
Source: Ecol Evol. 2022 Mar 23;12(3):e8751. doi: 10.1002/ece3.8751 (PMC8941502; doi:10.1002/ece3.8751)
Supplement: Supplementary file 1 — Supplementary Material [file ECE3-12-e8751-s001.pdf]

Supplementary Material

Benefits and limitations of a new genome-based PCR-RFLP genotyping assay (GB-RFLP): a SNP-based detection method for identification of species in extremely young adaptive radiations

Claudius F. Kratochwil[1,2], Andreas F. Kautt[1,3], Sina Rometsch[1] and Axel Meyer[1]

[1] Chair in Zoology and Evolutionary Biology, Department of Biology, University of Konstanz, Konstanz, Germany

[2] Present address: Institute of Biotechnology, HiLIFE, 00014 University of Helsinki, Finland

[3] Present address: Department of Organismic and Evolutionary Biology, Harvard University, Cambridge, MA, U.S.A.

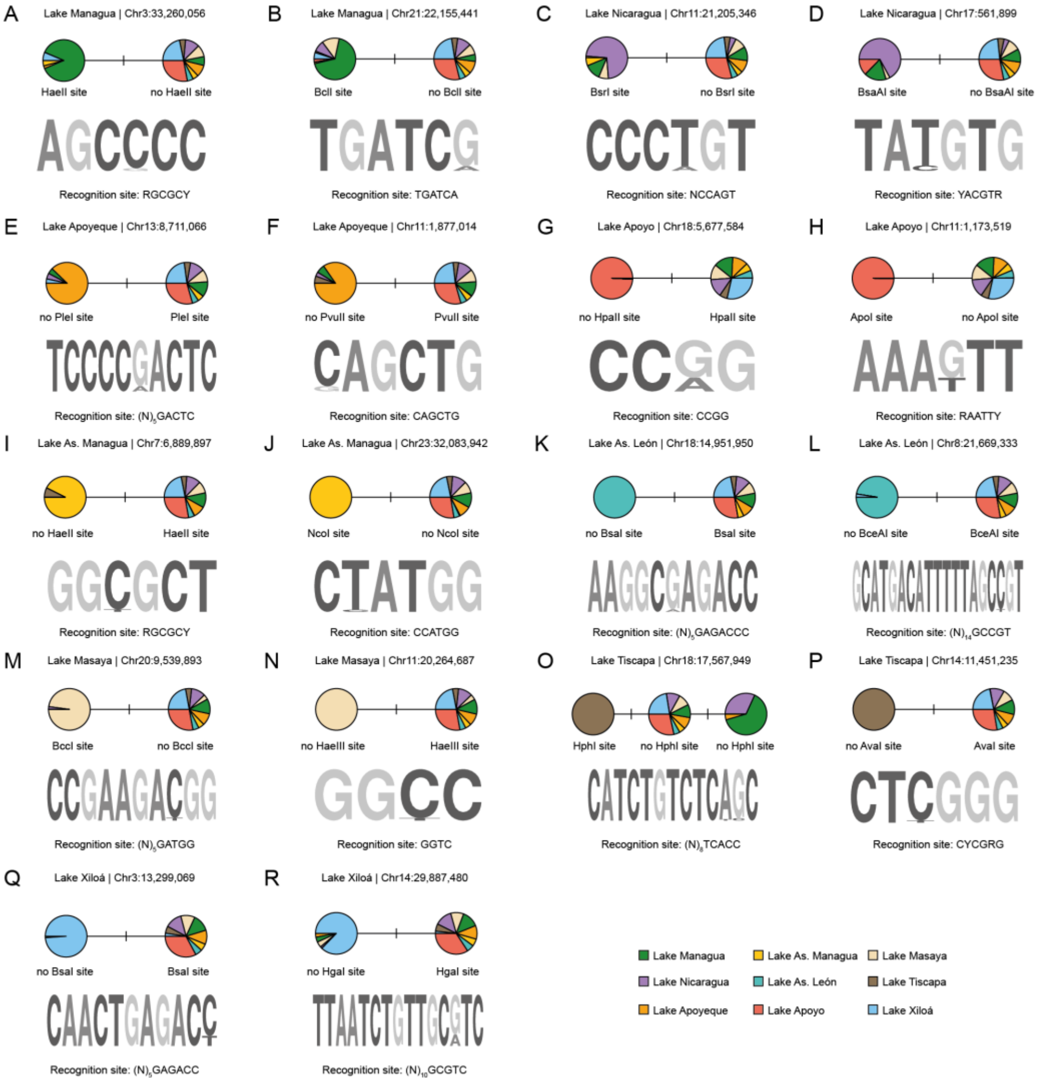

SUPPLEMENTARY FIGURE 1 Lake specific markers with haplotype networks (left side is always ingroup, right side outgroup, populations are color-coded) and allele frequency across the whole genomic dataset (shown as motif graph) for the restriction site.

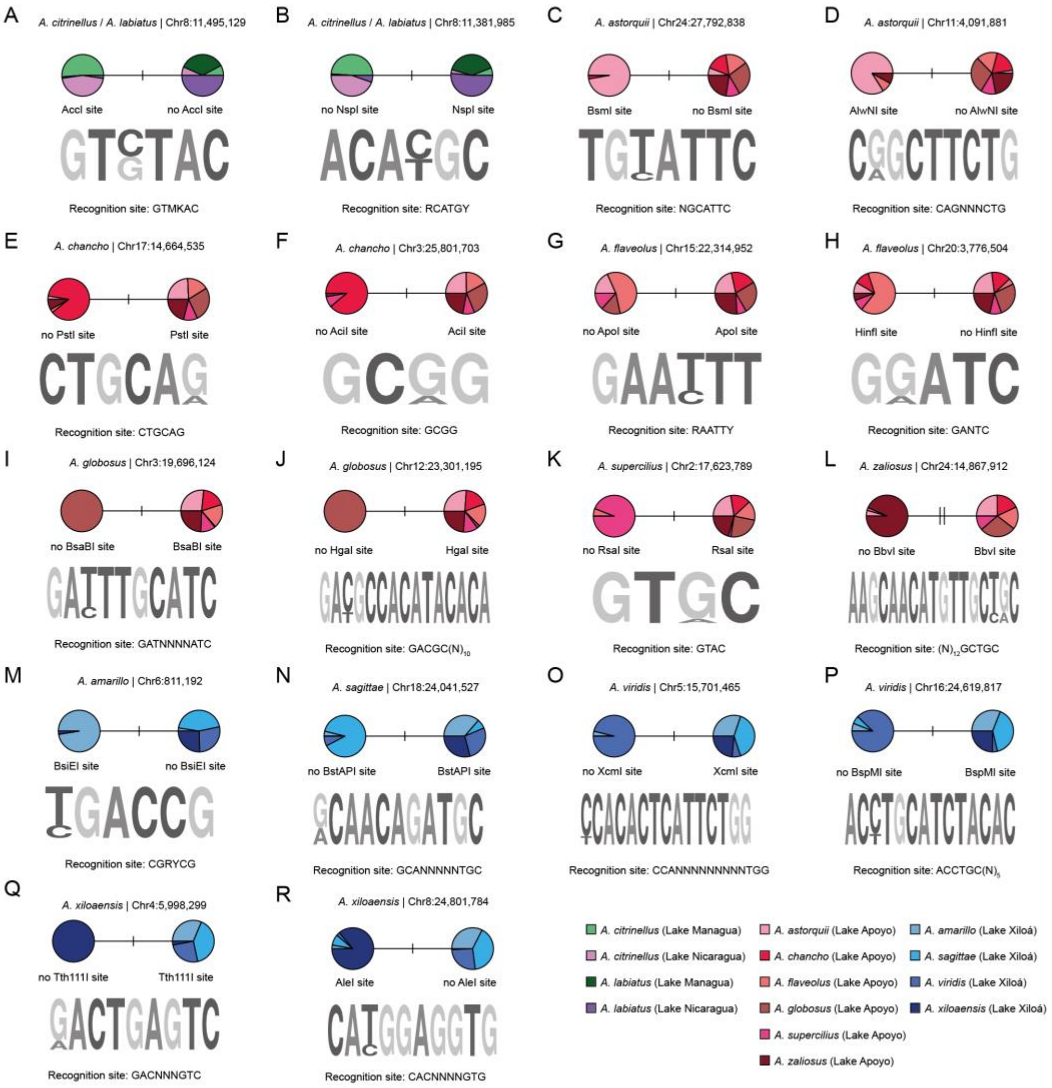

SUPPLEMENTARY FIGURE 2 Species-specific markers with haplotype networks (left side is always ingroup, right side outgroup; species are color-coded) and allele frequency across the whole genomic dataset (shown as motif graph) for the restriction site.

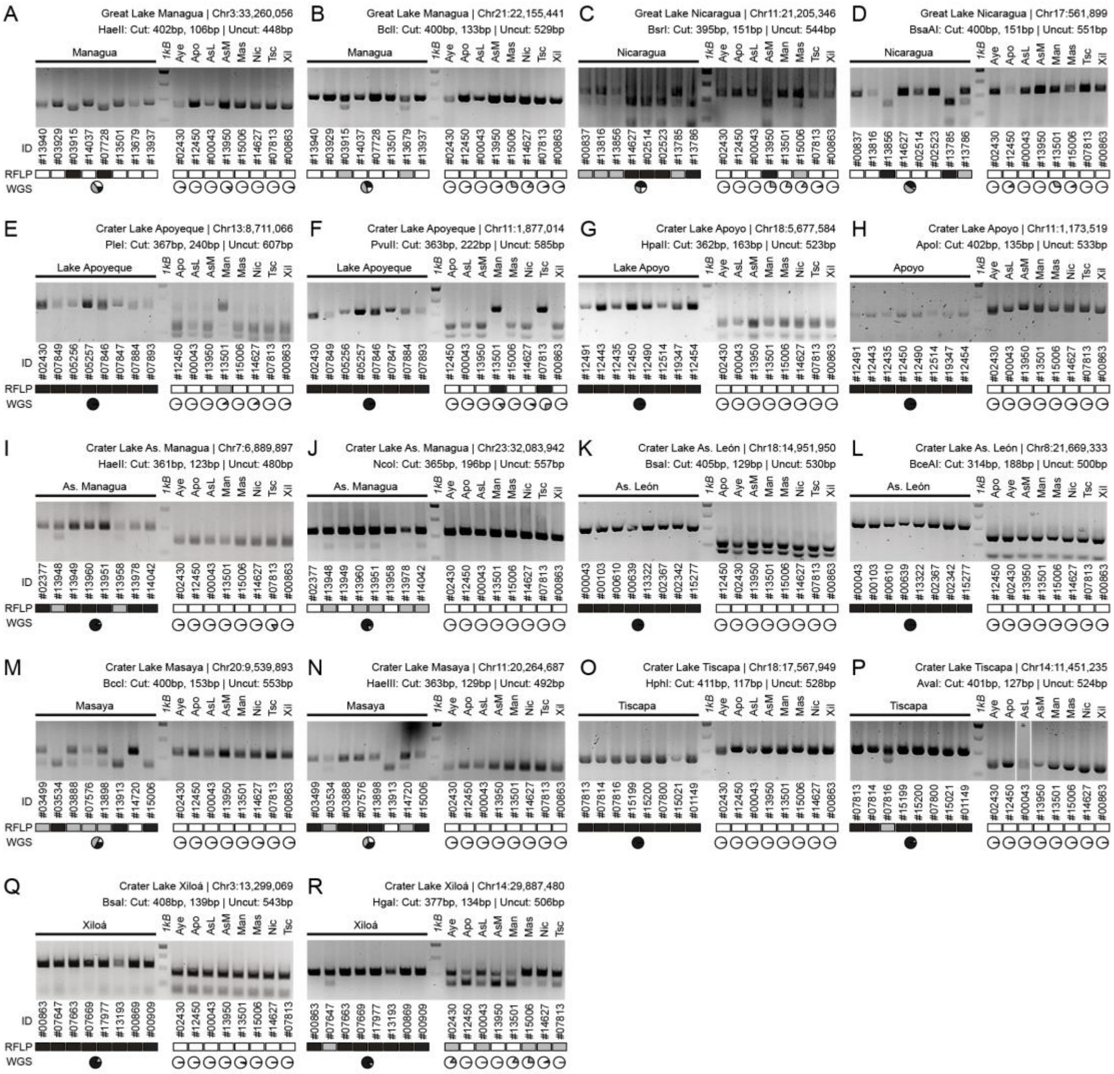

SUPPLEMENTARY FIGURE 3 Lake-specific GB-RFLP assays. Target population, genomic position of the RFLP, enzyme and fragment sizes are indicated in the title. We always used eight ingroup samples and eight outgroup samples. Black/Grey/White boxes indicate the genotype (black: homozygous for the common allele in the target population, grey: heterozygous, white: homozygous for the rare allele). Pie charts indicate how common the genotypes were in the whole genome re-sequencing (WGS) dataset for the respective populations. We always loaded all samples for direct comparison on the same gel (expect in P where one sample did not work and was repeated). Abbreviations: Aye: Apoyeque, Apo: Apoyo, AsL: Asososca León, AsM: Asososca Managua, Man: Managua, Mas: Masaya, Nic: Nicaragua, Tsc: Tiscapa, Xil: Xiloá, 1kB: 1kB marker.

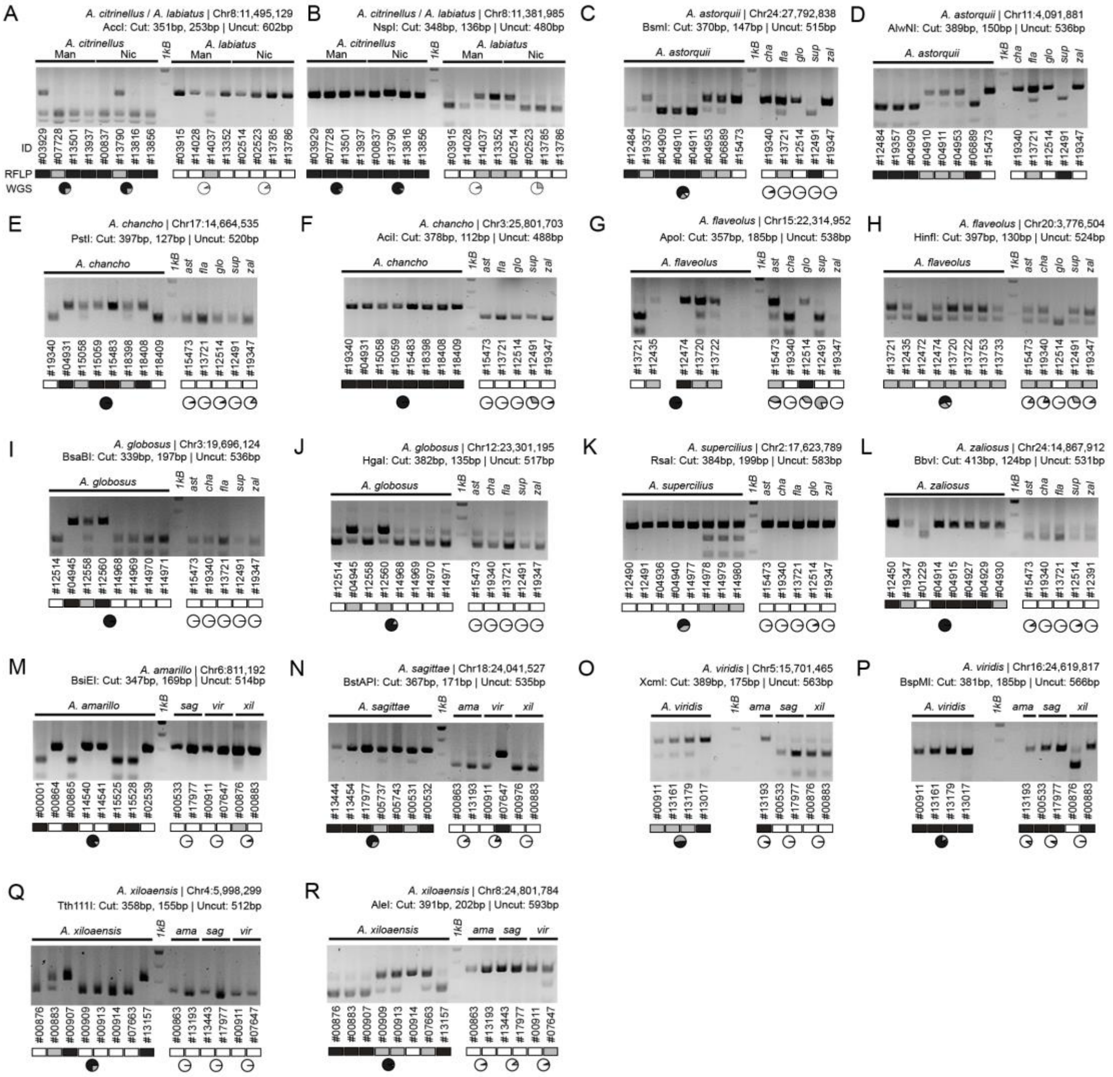

SUPPLEMENTARY FIGURE 4 Species-specific GB-RFLP assays for the great lake (A, B), Crater Lake Apoyo (C–L) and Crater Lake Xiloá species (M–R). Target population, genomic position of the RFLP, enzyme and fragment sizes are indicated in the title. Whenever possible (for some populations we had a limited amount of samples/tissue), we always used eight ingroup samples and eight outgroup samples. Black/Grey/White boxes indicate the genotype (black: homozygous for the common allele in the target population, grey: heterozygous, white: homozygous for the rare allele). Pie charts indicate how common the genotypes were found in the whole genome sequencing (WGS) dataset for the respective populations. We always loaded all samples for direct comparison on the same gel. Abbreviations: Man: Managua, Nic: Nicaragua, ast: *A. astorquii*, cha: *A. chancho*, fla: *A. flaveolus*, glo: *A. globosus*, sup: *A. supercilius*, zal: *A. zaliosus*, ama: *A. amarillo*, sag: *A. sagittae*, vir, *A. viridis*: xil: *A. xiloaensis*, 1kB: 1kB marker.

SUPPLEMENTARY TABLE 1 Primers and position of 5’ and 3’ binding sites in the genome.

Marker<br>(Test population | SNP Position)	Fwd. primer	Rev. primer	5'	3'
Lake Managua | Chr3:33,260,056	GAAAGCAGCAGGTAAGCCAC	ACGCCCTGATGTCTCTGAGTA	33259906	33260455
Lake Managua | Chr21:22,155,441	AGGCAGGCAAATGTCCAGAA	CATTTACGCTCACACACTGGG	22155308	22155836
Lake Nicaragua | Chr11:21,205,346	AGTGTGTTCTTTGTCTGCTGC	ATTTATACCTCTGCCTTCGGGC	21205198	21205741
Lake Nicaragua | Chr17:561,899	CACACACAGGATCATTCAAGCA	TGTGTCTCCTCTCCTGCTG	561749	562299
Lake Apoyeque | Chr13:8,711,066	CTGGAGGTCACCTAAAGGCA	CTGCCCAAACTTTCACACCG	8710822	8711428
Lake Apoyeque | Chr11:1,877,014	TTCCCTGACTGGACTGACAA	CCACAGAGTTGGGAGCATCA	1876795	1877379
Lake Apoyo | Chr18:5,677,584	TGCATTCAACCAAAATACCCT	CATGACCACACCACAAAGCA	5677422	5677944
Lake Apoyo | Chr11:1,173,519	CAGCAGCAGAATGAAAGCGG	TCTGGTGCATGCTGTTTATTTCTC	1173386	1173918
Lake As. Managua | Chr7:6,889,897	TAGACTACTGCCAGGCCCAT	TCAGCAAGGAAGTCTGACCC	6889777	6890256
Lake As. Managua | Chr23:32,083,942	GACGTCGGCCAGTCTCTTAC	GACTCTAGGAACACGCCTCG	32083581	32084137
Lake As. León | Chr18:14,951,950	GCACATGTCCAAACGCCAAA	AATGTGCCTGGTCAGTGTCA	14951820	14952349
Lake As. León | Chr8:21,669,333	TGAGCCAAAAAGGTGTAGGAC	TCTCCTTCATCCTGTGAGCAA	21669005	21669504
Lake Masaya | Chr20:9,539,893	AAGAGAGGAGCGACTTGCCT	TGTTTCTAGCTGGCTGGTCT	9539734	9540286
Lake Masaya | Chr11:20,264,687	ACTGAAACAGCTCCTCCCAC	TCCACACTCACTTAAATCTCCGT	20264558	20265049
Lake Tiscapa | Chr18:17,567,949	AGACTCAACAGCTCAATGCCT	GGACGTCATTTAGACCACATAGA	17567822	17568349
Lake Tiscapa | Chr14:11,451,235	AGAATCTCAAGTGAGGAAGGAGAC	CACACTGGACAGTGAATGGAGA	11451111	11451634
Lake Xiloá | Chr3:13,299,069	GCCTTTTTCGCTGTCACTGT	CCGTTGACACTGGTTAGCCT	13298924	13299466
Lake Xiloá | Chr14:29,887,480	CAGCTCAACTCCAGAGCCAA	TGCAGTTTAAGGTATCACTGTGT	29887339	29887844
A. citrinellus | Chr8:11,495,129	CTGTTGAGTTGGCGTCTGTG	GCTTGATACTGTCTCCCAGTG	11494878	11495479
A. citrinellus | Chr8:11,381,985	ATGGCCTCAGGTGTTTTCCT	ACTCCAAAGCTGCACTCACA	11381851	11382330
A. astorquii | Chr24:27,792,838	GGCTGAAAGCAAGCAACACA	GCATGTGGACTGTGGAAGGA	27792692	27793206
A. astorquii | Chr11:4,091,881	GCCGTAGCGATGTTTAGCCT	GCCGTCTATATTCCACCGCA	4091736	4092271
A. chancho | Chr17:14,664,535	GCCATGTGTGTTTCAGAGGC	GCACACAGAGACCTGCTTCA	14664408	14664927
A. chancho | Chr3:25,801,703	CTGCTCCAAGGCCCAGTATAA	GGGCTTGTTCACCCAAATCT	25801326	25801813
A. flaveolus | Chr15:22,314,952	CAGGGTGTAATGATTGTACAGCAC	GACCACCAAGGTAAACGCT	22314597	22315134
A. flaveolus | Chr20:3,776,504	GCAGCGTGATGTTCTCCTCA	GGAAAGCGCTCGTCACTG	3776377	3776900
A. globosus | Chr3:19,696,124	ACAAACCGCAGTTAGTCGCT	CGATTCAGCTGAGTGAAAACC	19695788	19696323
A. globosus | Chr12:23,301,195	CCACTGTGACTTCACCCACT	GCTGGGCTCAGCCTTAGA	23301068	23301584
A. supercilius | Chr2:17,623,789	CGACTTGTTTTTCTCCCAGTCA	GTGCAGCTCTATTGTCCCAA	17623405	17623987
A. zaliosus | Chr24:14,867,912	TGGAGTGAAAGGGTCAGAGG	ACAGCCCAGTGTGAAATGAC	14867779	14868309
A. amarillo | Chr6:811,192	GTCTTTGCTACGCTCTCCGT	ATAGGGCTGCAACGATTCCT	810849	811362
A. sagittae | Chr18:24,041,527	AACTCCATCCTGGAACACCG	CTTACTCTCCTTGGCAAGCAAAAA	24041167	24041701
A. viridis | Chr5:15,701,465	TCCTTGTGGAGCTGCTTTGT	TGGGTGAGAACAGAGGGGAT	15701084	15701646
A. viridis | Chr16:24,619,817	TATATTCACCGTGGGCCTGC	CTTCTGCTGAAAACCGTCGC	24619444	24620009
A. xiloaensis | Chr4:5,998,299	GGGACCTGTGGCTTTTTCCT	TCCTGTTCTCCCTCACCCA	5997946	5998457
A. xiloaensis | Chr8:24,801,784	AACGAAGGCTCACCTGACTC	GTTTCAGATTTGTGTGGCCTGT	24801585	24802177

SUPPLEMENTARY TABLE 2 Summary of genotype frequencies in ingroup (target population) and outgroup (all other populations assuming equal proportions). Genotype frequencies were calculated from allele frequencies of the individual populations assuming Hardy-Weinberg equilibrium. The probability for the ingroup is calculated by assuming that there is a 50:50 chance that an individual is from ingroup or outgroup and calculating the chance based on the relative genotype frequencies for a certain genotype to be from ingroup or outgroup (the probability to be in the outgroup are the difference to 100%)

Marker	Genotype freq. ingroup			Genotype freq. outgroup			Probability to be in ingroup		
(Test population | SNP Position)	AA	AB	BB	AA	AB	BB	AA	AB	BB
Lake Managua | Chr3:33,260,056	16.8	48.4	34.8	0	1.9	98.1	99.8	96.3	26.2
Lake Managua | Chr21:22,155,441	30.3	49.5	20.2	0.3	6.6	93.1	98.9	88.2	17.9
Lake Nicaragua | Chr11:21,205,346	36.5	47.8	15.7	0.4	7.3	92.3	98.9	86.7	14.5
Lake Nicaragua | Chr17:561,899	43.1	45.1	11.8	0.4	5.4	94.2	99.2	89.2	11.1
Lake Apoyeque | Chr13:8,711,066	97.5	2.5	0	0	2.3	97.6	100	51.3	0
Lake Apoyeque | Chr11:1,877,014	92.6	7.2	0.1	0.3	4.9	94.8	99.7	59.6	0.1
Lake Apoyo | Chr18:5,677,584	99.2	0.8	0	0	0.6	99.4	100	59.4	0
Lake Apoyo | Chr11:1,173,519	98.3	1.7	0	0	0.3	99.7	100	86.5	0
Lake As. Managua | Chr7:6,889,897	90.2	9.5	0.3	0.1	1.7	98.2	99.9	84.6	0.3
Lake As. Managua | Chr23:32,083,942	76.6	21.9	1.6	0	0	100	100	100	1.5
Lake As. León | Chr18:14,951,950	100	0	0	0	0	100	100	NA	0
Lake As. León | Chr8:21,669,333	100	0	0	0	0.1	99.9	100	0	0
Lake Masaya | Chr20:9,539,893	33.1	48.9	18.1	0	0.3	99.7	100	99.5	15.3
Lake Masaya | Chr11:20,264,687	28.9	49.7	21.4	0	0	100	100	100	17.6
Lake Tiscapa | Chr18:17,567,949	100	0	0	0	0	100	100	NA	0
Lake Tiscapa | Chr14:11,451,235	90.2	9.5	0.3	0	0	100	100	100	0.2
Lake Xiloá | Chr3:13,299,069	82.9	16.3	0.8	0	0.5	99.5	100	97.1	0.8
Lake Xiloá | Chr14:29,887,480	87.8	11.8	0.4	0.3	6.8	92.9	99.6	63.4	0.4
A. citrinellus / *A. labiatus* | Chr8:11,495,129	91.4	8.4	0.2	1.4	20.7	78	98.5	29	0.2
A. citrinellus / *A. labiatus* | Chr8:11,381,985	83.1	16.1	0.8	0.4	12	87.6	99.5	57.4	0.9
A. astorquii | Chr24:27,792,838	57.9	36.4	5.7	0	1.2	98.8	100	96.8	5.5
A. astorquii | Chr11:4,091,881	83.4	15.9	0.8	0.5	7.8	91.7	99.4	67	0.8
A. chancho | Chr17:14,664,535	100	0	0	0.1	4.7	95.2	99.9	0	0
A. chancho | Chr3:25,801,703	100	0	0	0.5	6	93.5	99.5	0	0
A. flaveolus | Chr15:22,314,952	100	0	0	5	22.8	72.2	95.2	0	0
A. flaveolus | Chr20:3,776,504	51.7	40.4	7.9	0.9	13.7	85.4	98.3	74.7	8.5
A. globosus | Chr3:19,696,124	96	3.9	0	0	0	100	100	100	0
A. globosus | Chr12:23,301,195	84.6	14.7	0.6	0	0	100	100	100	0.6
A. supercilius | Chr2:17,623,789	64	32	4	0	1.2	98.8	100	96.4	3.9
A. zaliosus | Chr24:14,867,912	100	0	0	0.1	2.9	97.1	99.9	0	0
A. amarillo | Chr6:811,192	86.2	13.3	0.5	0	2	97.9	100	86.8	0.5
A. sagittae | Chr18:24,041,527	72.6	25.2	2.2	0.5	9.9	89.5	99.3	71.7	2.4
A. viridis | Chr5:15,701,465	54.1	38.9	7	0	1.5	98.4	100	96.2	6.6
A. viridis | Chr16:24,619,817	72.8	25.1	2.2	0.1	5.4	94.5	99.8	82.3	2.2
A. xiloaensis | Chr4:5,998,299	76.6	21.9	1.6	0	0	100	100	100	1.5
A. xiloaensis | Chr8:24,801,784	93.8	6.1	0.1	0.2	7	92.9	99.8	46.6	0.1

SUPPLEMENTARY TABLE 3 Summary of the percentages of false positives, false negatives and correctly assigned samples for a bootstrapping dataset and the GB-RFLP dataset.

Marker	Based on genomic dataset			GB-RFLP		
(Test population | SNP Position)	False positives (%)	False negatives (%)	Percent correct (%)	False positives (%)	False negatives (%)	Percent correct (%)
Lake Managua | Chr3:33,260,056	34.1	1.9	81.7	0	75	62.5
Lake Managua | Chr21:22,155,441	19.7	6.9	86.5	0	75	62.5
Lake Nicaragua | Chr11:21,205,346	16	7.5	88.3	25	0	87.5
Lake Nicaragua | Chr17:561,899	11.9	5.6	91.2	0	62.5	68.75
Lake Apoyeque | Chr13:8,711,066	0	2.5	98.8	12.5	0	93.75
Lake Apoyeque | Chr11:1,877,014	0.2	5.3	97.3	25	0	87.5
Lake Apoyo | Chr18:5,677,584	0	0.7	99.7	0	0	100
Lake Apoyo | Chr11:1,173,519	0	0.3	99.8	0	0	100
Lake As. Managua | Chr7:6,889,897	0.3	1.6	99	0	0	100
Lake As. Managua | Chr23:32,083,942	1.5	0	99.2	0	37.5	81.25
Lake As. León | Chr18:14,951,950	0	0	100	0	0	100
Lake As. León | Chr8:21,669,333	0	0	100	0	0	100
Lake Masaya | Chr20:9,539,893	17.7	0.4	90.8	0	12.5	93.75
Lake Masaya | Chr11:20,264,687	21.9	0	89.3	0	12.5	93.75
Lake Tiscapa | Chr18:17,567,949	0	0	100	0	0	100
Lake Tiscapa | Chr14:11,451,235	0.3	0	99.8	0	0	100
Lake Xiloá | Chr3:13,299,069	0.8	0.5	99.4	0	0	100
Lake Xiloá | Chr14:29,887,480	0.4	7	96.3	62.5	0	68.75
A. citrinellus / *A. labiatus* | Chr8:11,495,129	8.4	1.2	95	0	25	87.5
A. citrinellus / *A. labiatus* | Chr8:11,381,985	0.8	12.4	93.4	37.5	0	81.25
A. astorquii | Chr24:27,792,838	5.4	1.1	96.5	40	12.5	73.75
A. astorquii | Chr11:4,091,881	0.7	7.9	95.4	40	12.5	73.75
A. chancho | Chr17:14,664,535	0	0.1	100	0	50	75
A. chancho | Chr3:25,801,703	0	0.6	99.8	0	0	100
A. flaveolus | Chr15:22,314,952	0	4.9	97.5	20	80	50
A. flaveolus | Chr20:3,776,504	7.7	14.7	88.8	80	12.5	53.75
A. globosus | Chr3:19,696,124	0	0	100	0	62.5	68.75
A. globosus | Chr12:23,301,195	0.6	0	99.7	0	75	62.5
A. supercilius | Chr2:17,623,789	4.2	1.4	97.4	0	62.5	68.75
A. zaliosus | Chr24:14,867,912	0	0.1	100	0	37.5	81.25
A. amarillo | Chr6:811,192	0.5	1.9	98.7	16.7	50	66.65
A. sagittae | Chr18:24,041,527	2	10.7	93.7	16.7	0	91.65
A. viridis | Chr5:15,701,465	6.9	1.7	95.7	20	0	90
A. viridis | Chr16:24,619,817	2.1	5.6	96.1	80	0	60
A. xiloaensis | Chr4:5,998,299	1.7	0	99.2	0	62.5	68.75
A. xiloaensis | Chr8:24,801,784	5.9	0.3	96.8	0	50	75

SUPPLEMENTARY TABLE 4 Number of correctly assigned samples using individual markers or both markers combined.

Test population	Marker 1 (%)	Marker 2 (%)	Markers combined (%)
Managua	62.5	62.5	68.75
Nicaragua	87.5	68.75	81.25
Apoyeque	93.75	87.5	93.75
Apoyo	100	100	100
As. Managua	100	81.25	93.75
As. León	100	100	100
Masaya	93.75	93.75	100
Tiscapa	100	100	100
Xiloá	100	68.75	100
A. citrinellus / A. labiatus	87.5	81.25	100
A. astorquii	73.75	73.75	73.75
A. chancho	75	100	75
A. flaveolus	50	60	50
A. globosus	68.75	62.5	68.75
A. viridis	60	90	90
A. xiloaensis	68.75	75	75
